# *C9orf72* gene networks in the human brain correlate with cortical thickness in C9-FTD and implicate vulnerable cell types

**DOI:** 10.3389/fnins.2024.1258996

**Published:** 2024-02-26

**Authors:** Iris J. Broce, Daniel W. Sirkis, Ryan M. Nillo, Luke W. Bonham, Suzee E. Lee, Bruce L. Miller, Patricia A. Castruita, Virginia E. Sturm, Leo S. Sugrue, Rahul S. Desikan, Jennifer S. Yokoyama

**Affiliations:** ^1^Memory and Aging Center, Department of Neurology, Weill Institute for Neurosciences, University of California San Francisco, San Francisco, CA, United States; ^2^Department of Neurosciences, University of California San Diego, San Diego, CA, United States; ^3^Department of Radiology and Biomedical Imaging, University of California San Francisco, San Francisco, CA, United States; ^4^Global Brain Health Institute, University of California San Francisco, San Francisco, CA, United States

**Keywords:** C9orf72, amyotrophic lateral sclerosis, frontotemporal dementia, imaging transcriptomics, gene networks, neurodegeneration

## Abstract

**Introduction:**

A hexanucleotide repeat expansion (HRE) intronic to chromosome 9 open reading frame 72 (*C9orf72*) is recognized as the most common genetic cause of amyotrophic lateral sclerosis (ALS), frontotemporal dementia (FTD), and ALS-FTD. Identifying genes that show similar regional co-expression patterns to *C9orf72* may help identify novel gene targets and biological mechanisms that mediate selective vulnerability to ALS and FTD pathogenesis.

**Methods:**

We leveraged mRNA expression data in healthy brain from the Allen Human Brain Atlas to evaluate *C9orf72* co-expression patterns. To do this, we correlated average *C9orf72* expression values in 51 regions across different anatomical divisions (cortex, subcortex, and cerebellum) with average gene expression values for 15,633 protein-coding genes, including 54 genes known to be associated with ALS, FTD, or ALS-FTD. We then performed imaging transcriptomic analyses to evaluate whether the identified *C9orf72* co-expressed genes correlated with patterns of cortical thickness in symptomatic *C9orf72* pathogenic HRE carriers (*n* = 19) compared to controls (*n* = 23). Lastly, we explored whether genes with significant *C9orf72* imaging transcriptomic correlations (i.e., “*C9orf72* imaging transcriptomic network”) were enriched in specific cell populations in the brain and enriched for specific biological and molecular pathways.

**Results:**

A total of 2,120 genes showed an anatomical distribution of gene expression in the brain similar to *C9orf72* and significantly correlated with patterns of cortical thickness in *C9orf72* HRE carriers. This *C9orf72* imaging transcriptomic network was differentially expressed in cell populations previously implicated in ALS and FTD, including layer 5b cells, cholinergic neurons in the spinal cord and brainstem and medium spiny neurons of the striatum, and was enriched for biological and molecular pathways associated with protein ubiquitination, autophagy, cellular response to DNA damage, endoplasmic reticulum to Golgi vesicle-mediated transport, among others.

**Conclusion:**

Considered together, we identified a network of *C9orf72* associated genes that may influence selective regional and cell-type-specific vulnerabilities in ALS/FTD.

## Introduction

Frontotemporal dementia (FTD) and amyotrophic lateral sclerosis (ALS) are neurodegenerative disorders that have overlapping clinical, genetic, and neuropathological features. FTD is the most common form of dementia diagnosed in people younger than 65 years old and is characterized by changes in social behavior and/or language abilities due to neurodegeneration of the frontal and temporal lobes. Depending on the signs and symptoms, FTD patients are classified into one of three different syndromes: behavioral variant FTD (bvFTD) or one of two forms of primary progressive aphasias (PPA), including non-fluent variant PPA (nfvPPA) and semantic variant PPA (svPPA). ALS is the most common form of adult-onset motor neuron disease (MND) and is characterized by progressive degeneration of both upper motor neurons of the motor cortex and lower motor neurons of the brainstem and spinal cord at disease onset. Although motor neuron damage predominates in ALS, other neuronal populations including within frontal, temporal, and parietal cortical circuits, the basal ganglia, and dorsal root ganglia are also involved in some patients (Rosness et al., 2008; Rabinovici and Miller, 2010; van Vliet et al., 2013; Bang et al., 2015). Although the clinical phenotypes of FTD and ALS can be heterogeneous, about 15% of people with bvFTD, 11% of patients with nfvPPA, and 19% of patients with svPPA may eventually develop motor symptoms consistent with ALS (Rascovsky et al., 2011; Vinceti et al., 2019). Similarly, about 50% of ALS patients develop cognitive and behavioral impairment, with 13% meeting diagnostic criteria for bvFTD (Ljubenkov and Miller, 2016). This clinical overlap may, at least in part, be due to shared neuropathology due to aggregation of TDP-43, which drives MND and around half of frontotemporal lobar degeneration (FTLD) pathology. After decades of research, it is now recognized that a pathogenic hexanucleotide repeat expansion (HRE) intronic to chromosome 9 open reading frame 72 (*C9orf72*) is the most common genetic cause of ALS, FTD, and ALS combined with FTD (ALS-FTD) (DeJesus-Hernandez et al., 2011; Renton et al., 2011).

The clinical syndromes of FTD and ALS represent the manifestations of underlying neuropathology that results in the dysfunction and death of neurons in specific neuroanatomical regions. For example, individuals with ALS display muscle weakness and wasting because of dysfunction and death of upper and lower motor neurons. *C9orf72*-FTD typically manifests as bvFTD (Vatsavayai et al., 2019), and anatomically, the cortico-striato-thalamic network (Lee et al., 2014), and medial pulvinar thalamus, specifically, appear to be the primary structures affected (Sha et al., 2012; Yokoyama et al., 2014; Vatsavayai et al., 2016; Bonham et al., 2023). Understanding the genetic landscape of normal *C9orf72* - that is, genes that are normally co-expressed with non-expanded *C9orf72* - may clarify why certain brain regions are selectively targeted in ALS or FTD (henceforth, ALS/FTD), why some patients may be more likely to develop either ALS/FTD, or both; and which cell-type populations and biological mechanisms are involved.

There is considerable genetic overlap between ALS and FTD. Beyond *C9orf72*, pathogenic variants in *TARDBP, SQSTM1, VCP, FUS, TBK1, CHCHD10*, and *UBQLN2* (Abramzon et al., 2020) are also closely associated with both diseases. Notably, ALS, FTD, and ALS-FTD patients carrying pathogenic HRE in *C9orf72* sometimes carry a second gene mutation previously implicated in ALS or FTD (Gijselinck et al., 2018). Multiple gene abnormalities in *C9orf72* HRE carriers have been detected in *TARDBP* (Chio et al., 2012; Cooper-Knock et al., 2012; van Blitterswijk et al., 2012), *TBK1* (Van Mossevelde et al., 2016), *FUS* (Millecamps et al., 2012; van Blitterswijk et al., 2012), *SOD1* (Millecamps et al., 2012; van Blitterswijk et al., 2012), *OPTN* (Cooper-Knock et al., 2012; Millecamps et al., 2012), *ANG* (Millecamps et al., 2012), *UBQLN2* (Millecamps et al., 2012), *DAO* (Millecamps et al., 2012), *GRN* (Ferrari et al., 2012), *SQSTM1* (Almeida et al., 2016), and *PSEN2* (Cooper-Knock et al., 2012; Ferrari et al., 2012; Van Mossevelde et al., 2016). Thus, the possibility of carrying a *C9orf72* HRE and a second ALS/FTD pathogenic variant is likely not random. Specific ALS/FTD genes may be co-expressed and form a functional network in brain regions that are selectively vulnerable to ALS and FTD. Consequently, disruption of these genes, depending on the affected neuroanatomical regions, is likely to influence ALS/FTD-related disease processes. Identifying genes that show similar regional co-expression patterns as *C9orf72* may, therefore, help identify novel gene targets, pathways, and biological mechanisms that mediate selective vulnerability to ALS/FTD pathogenesis.

To explore the neuroanatomical basis of shared genetic risk in the FTD/ALS spectrum, we performed gene co-expression analysis to identify genes that show regional co-expression patterns similar to *C9orf72*, the most common shared genetic contributor to ALS/FTD. We then implemented an imaging transcriptomics approach to evaluate whether the identified *C9orf72* co-expressed genes also correlate with patterns of cortical thickness in symptomatic *C9orf72* expansion carriers. Lastly, we evaluated whether certain cell populations within the brain may be selectively vulnerable to ALS/FTD pathogenesis.

## Materials and methods

### Gene expression in the adult human brain

We investigated co-expression and regional patterns of gene expression in the healthy brain across the cortex, subcortex, and cerebellum using microarray gene expression data from the Allen Human Brain Atlas (AHBA)^1^, a publicly available microarray dataset widely used for exploration of gene networks in the human brain (Hawrylycz et al., 2012). The microarray data was sampled from six adult human donors (3 White, 2 African American, 1 Hispanic, aged 24−57 years) in roughly 500 tissue samples from each donor, either in the left hemisphere only (*n* = 4) or in both hemispheres (*n* = 2). Although the same anatomical regions were sampled in all six donors, both the exact number and position of samples varied among donors. Donors also differed in other characteristics, including cause of death, post-mortem intervals, brain pH, tissue cytoarchitectural integrity, RNA quality, and number of probes used for each gene. Given space constraints, we refer the reader to the original technical white paper for additional details regarding the dissection methods, quality control, and normalization measures taken.^2^

The AHBA data were preprocessed and mapped to parcellated brain regions using a publicly available “abagen” processing pipeline.^3^ We applied the recommended default parameters outlined in the original manuscripts (Arnatkeviciute et al., 2019; Markello et al., 2021). Briefly, all available probes (Custom and Agilent) were included in the analyses. Probes that did not exceed background noise in at least 50% of all cortical and subcortical samples across all subjects were excluded. As more than one probe can be available for a single gene, the probe with the higher differential stability score was selected (default parameter). Probe selection was performed for each donor separately. The Montreal Neurological Institute (MNI) coordinates of tissue samples were updated to those generated via non-linear registration using Advanced Normalization Tools (ANTs). Tissue samples were assigned to brain regions in the provided atlas if their MNI coordinates were within 2 mm of a given parcel. To reduce potential misassignment, sample-to-region matching was constrained by hemisphere and gross anatomical divisions (cortex, subcortex/brainstem, and cerebellum). All tissue samples not assigned to a brain region in the provided atlas were discarded. We used the Desikan “aparcaseg” atlas (34 nodes per hemisphere + subcortex) to map tissue samples to cortical and subcortical regions^4^ (Desikan et al., 2006). We used the Diedrichsen atlas to map tissue samples to cerebellar regions^5^ (Diedrichsen et al., 2009). Inter-subject variation was addressed by normalizing tissue sample expression values across genes using a robust sigmoid function. Normalized expression values were then rescaled to the unit interval. Gene expression values across genes were normalized separately for each anatomic division (cortex, subcortex, and cerebellum) also using a robust sigmoid function. Samples assigned to the same region were averaged separately for each donor and then across donors. Gene expression values for the same region and gene sampled from both hemispheres from the same donor were averaged.

**FIGURE 1:**
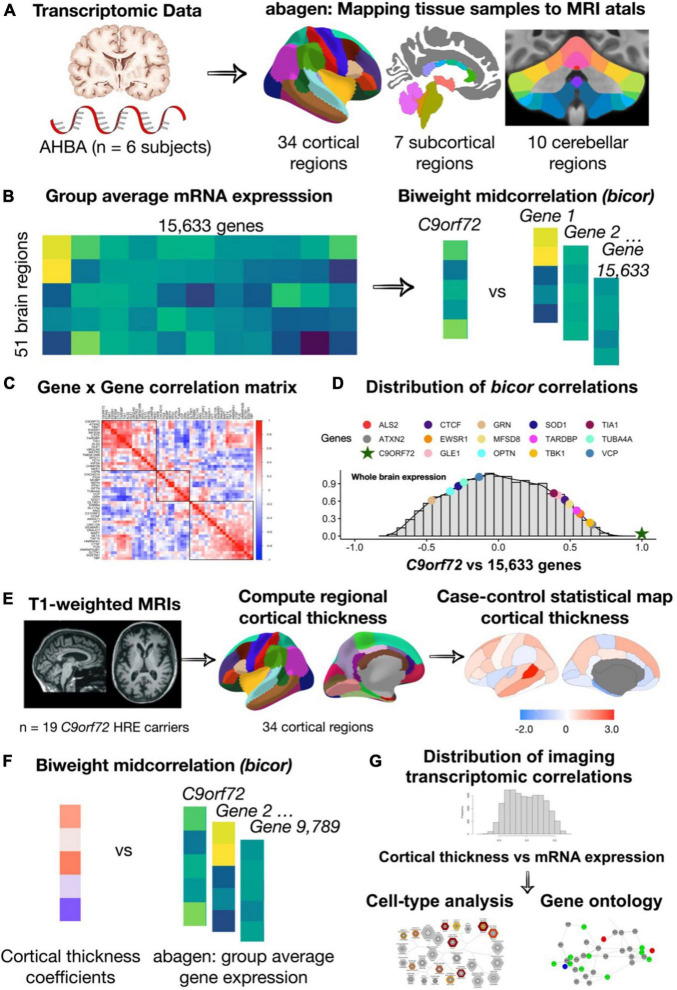
An overview of the methodology used for mapping mRNA gene expression to MRI atlas, estimation of regional gene expression, co-expression gene analysis, gene ontological and cell type analyses. **(A)** Allen Human Brain Atlas samples of gene expression data were mapped to the 51 brain regions (34 cortical regions, 7 subcortical regions, and 10 cerebellar regions) according to the anatomical parcellations. **(B)** Samples across the left/right hemisphere for the same region and gene were averaged across donor resulting in average gene expression values for 15,633 protein-coding genes. **(C)** Using the biweight correlation method, we separately correlated the columns of each expression matrix to generate a 54 × 54 ALS/FTD gene co-expression matrix. **(D)** We also used the biweight correlation method to correlate average *C9orf72* expression values for each region with average expression of all available 15,633 protein-coding genes. Subsequent analysis focused on *C9orf72*-associated genes, defined as having a correlation value above the absolute value of 0.5. **(E)** Mean cortical thickness were from 34 cortical regions for patients with *C9orf72* hexanucleotide repeat expansions (*HRE*) compared to controls. **(F)** Using the biweight correlation method, we correlated the case-control cortical thickness coefficients for each region with average regional expression values for each *C9orf72*-associated gene. **(G)** Genes that were significant after permutation testing underwent gene ontological analyses for biological processes and cell-type enrichment analyses.

After implementing the pre-processing and quality control steps outlined above (Figure 1A), gene expression values from 15,633 protein-coding genes from the six donors were included in the analyses. Gene expression values were computed for 51 brain regions (34 cortical regions, 7 subcortical regions, and 10 cerebellar regions).

### Independent gene expression sample validation

Median expression values (Log10 transcripts per million) for 10 distinct brain regions (amygdala, anterior cingulate cortex, caudate, cerebellar hemisphere, cerebellum, cortex, frontal cortex, hippocampus, nucleus accumbens, putamen, and substantia nigra) were obtained from the Genotype-Tissue Expression (GTEx) project database for the purpose of independent sample validation. Despite this dataset having lower spatial resolution compared to AHBA, we conducted a general comparison of co-expression patterns across the available regions for genes of interest to substantiate the generalizability of the AHBA expression data. We performed a Mantel test with permutations to examine whether the observed correlation between the two co-expression matrices was statistically significant. The permutation process involved shuffling the data multiple times to establish a null distribution, against which the actual correlation could be compared. *P*-values were calculated based on how often correlations in the permuted data are observed that are as extreme as, or more extreme than, the observed correlation.

### Symptomatic *C9orf72* HRE carriers

Nineteen symptomatic *C9orf72* HRE carriers participated in this study (10 males, 9 females; age range = 48−81 years, mean = 64 years, SD = 10 years). Individuals were recruited from the UCSF Memory and Aging Center (MAC) FTD cohort. *C9orf72* HRE carriers were clinically diagnosed with FTD (*n* = 8), and FTD-ALS/ALS (*n* = 5), mild cognitive impairment (*n* = 4), and other (*n* = 2). For the two patients categorized as other, both showed concerns for motor neuron disease and possibly FTD. Healthy controls included 23 related family members (6 males, 17 females; age range = 28−72 years, mean = 47 years, SD = 13 years). Detailed information on participant inclusion criteria can be found in prior reports (Lee et al., 2014; Bonham et al., 2023). The UCSF Committee on Human Research approved the procedures for all participants. All participants or their surrogates provided informed consent prior to participation.

### Mutation screening and genotyping

Genomic DNA was extracted from whole blood according to standard procedures. Participants were identified as carrying a pathogenic HRE in *C9orf72* if they harbored >30 hexanucleotide repeats (DeJesus-Hernandez et al., 2011). Participants in this study were negative for pathogenic variants in *MAPT* and *GRN*.

### MRI processing

All MRI scans were acquired on a 3T MRI scanner at the Neuroscience Imaging Center at UC San Francisco using previously described sequences (Bettcher et al., 2012). A high resolution T1-weighted image was acquired for all participants for structural reference, for the purpose of normalization, and for deriving morphological measures. MRI scans were processed using the FreeSurfer software package, version 6.0 (see text footnote 4) (Fischl, 2012). All images were visually inspected for segmentation accuracy and corrected as needed. We quantified disease burden using morphological measures of cortical thickness, since it provides a more sensitive measure of atrophy than gray matter volume (Winkler et al., 2010; Broce et al., 2023). For participants with multiple scans, the earliest scan with the best quality was selected. For visualization purposes, cortical brain images were generated using the R software statistical package “ggseg,” which allows for plotting brain atlases using simple features.

## Statistical analyses

### Statistical analysis of gene expression data

The R statistical package (version 4.2.2) was used for all statistical analysis. We first evaluated how average *C9orf72* expression in each brain region varied across different anatomical divisions: cortex, subcortex, and the cerebellum. One sample *t*-tests (two-tailed) were conducted to assess which of the 51 brain regions from the six donor samples expressed mRNA to a significantly greater or lesser degree compared to average mRNA expression across the whole brain. To correct for multiple tests, reported *p*-values were Holm–Bonferroni adjusted. Cohen’s d values for one-sample *t*-tests were calculated to yield a measure of effect size.

We then evaluated *C9orf72* co-expression patterns by correlating average *C9orf72* expression values for each region with average expression values from 54 other genes known to cause ALS, FTD, or combined ALS-FTD (Table 1). These genes were selected based on prior reports (Abramzon et al., 2020; Kirola et al., 2022). Two ALS/FTD genes, *PRPH* (encoding Peripherin), and *DAO* (encoding D-amino-acid oxidase), were excluded from analyses at the preprocessing stage due to low quality or coverage. We separately correlated the columns of each expression matrix to generate a 54 × 54 gene co-expression matrix, reflecting the relative expression patterns across cortical, subcortical, and cerebellar regions for each gene pair (Figures 1B, C). We used the biweight midcorrelation as the similarity measure. In biweight midcorrelation gene expression, values that are much higher or much lower than the median value are given less weight in the correlation calculation, which makes the method more robust to extreme values and outliers (Langfelder and Horvath, 2008). Clusters were identified to assess co-expression patterns using the complete linkage method (Murtagh and Contreras, 2017).

**TABLE 1:** These genes were selected based on prior reports (Abramzon et al., 2020; Kirola et al., 2022).

	ALS-FTD	ALS	FTD
Genetic mutations	*ANG, C9orf72, CCNF, CHCHD10, CHMP2B, FUS, hnRNPA1/A2B1, HTT repeat expansions, MATR3, OPTN, SQSTM1, SMCR8, TARDBP, TBK1, TREM2, TYROBP, UBQLN2, VCP, WDR41*	*Alsin, ANG, ANXA11, ATXN2, C21orf2, C9orf72, CHMP2B, DCTN1, DAO, ERBB4, EWSR1, FIG4, FUS, GLE1, GLT8D1, hnRNPA1, hnRNPA2B1, KIF5A, MATR3, NEK1, NFH, OPTN, Peripherin, PFN1, PPARGC1A, SETX, SIGMAR1, SOD1, SPG11, TARDBP, TAF15, TIA1, TUBA4A, UBQLN2, VAPB, VCP, TBK1.*	*CCNF, C9orf72, CHCHD10, CHMP2B, CTCF, DCTN1, GRN, hnRNPA1, hnRNPA2B1, MAPT, MATR3, MFSD8, SQSTM1, TBP, TBK1, VCP.*

Subsequently, to screen in an unbiased manner for novel genes that are co-expressed with *C9orf72*, we used the identical procedure described above. We correlated average *C9orf72* expression values for each region with average expression of all available 15,633 protein-coding genes (Figures 1B–D). To explore whether *C9orf72* co-expression patterns were driven by gene expression differences among the different anatomical divisions (cortex, subcortex, and cerebellum), we repeated the correlation analyses, separately, for each anatomical division. To reduce the influence of non-informative genes on subsequent analyses, genes with correlation values lower than an absolute value of 0.5 were excluded.

### Imaging transcriptomics analyses

A main goal of this study was to identify a network of genes or gene products that may contribute to the regional vulnerability of the human brain to *C9orf72* HRE-mediated pathology and, more generally, risk for ALS and FTD. Therefore, we identified correlations between average mRNA expression for each of the *C9orf72*-co-expressed genes from AHBA and differences in average cortical thickness from symptomatic *C9orf72* HRE carriers compared to controls. Since age and sex are known to affect cortical thickness, and the age range within *C9orf72* HRE carriers and controls was wide, our analyses controlled for age and sex. We followed established practices in the context of brain disorders to conduct these imaging transcriptomics analyses (Arnatkeviciute et al., 2022). In particular, our approach involved fitting separate linear models for each brain region to extract the estimated coefficients for case-control status on cortical thickness (i.e., *C9orf72* HRE carriers versus controls), while also controlling for age and sex. We then correlated the coefficients obtained from the linear models for each region with gene expression across the same regions. To evaluate the significance of these correlations, we performed a permutation test with 10,000 iterations. This approach determines whether the observed imaging transcriptomics correlations differ significantly from what might occur due to chance.

### Pathway enrichment analysis

We performed pathway enrichment analysis using the R statistical package “pathfindR” (Ulgen et al., 2019). Since enrichment analysis of a list of significant genes alone may not be informative enough to explain underlying disease mechanisms, we used “pathfindR,” which leverages interaction information from a protein-protein interaction network (PIN) to identify distinct active subnetworks and then perform enrichment analyses on these subnetworks. We preformed pathway enrichment analysis for three gene sets: “KEGG,” “GO-BP,” and “GO-MF” (all for *Homo sapiens*). For visualization, we plotted the top 30 terms for each gene set based on *p*-value.

## Results

### *C9orf72* gene expression patterns in the brain

**FIGURE 2:**
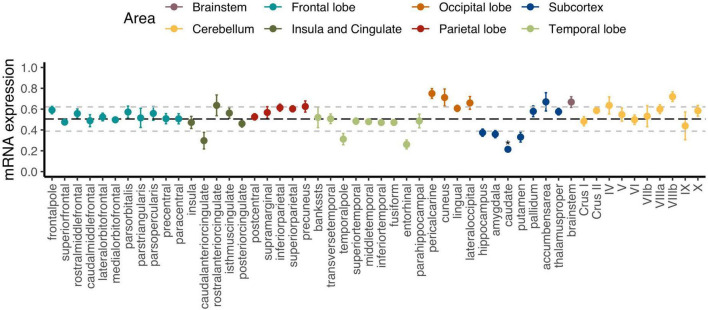
Regional *C9orf72* gene expression in the human brain. Each point represents mean expression from six donors with standard errors for a given brain region. Black bolded dashed line represents the mean expression across all genes with 1 standard deviation (± ) also shown in light gray dashed lines. *Adjusted *p* < 0.05.

Figure 2 displays how regional *C9orf72* expression varies across the cortex, subcortex, and the cerebellum. The caudate was the only brain region that was significantly different from average expression across the whole brain after Holm–Bonferroni correction (*p*-adjusted = 0.006). The full results can be found in Supplementary Table 1. Group-level analysis tends to obscure how expression and imaging markers co-vary across regions within individual participants (Broce et al., 2023). In our subsequent analyses, we directly measure the strength of regional correlations at the individual-participant level, thus avoiding the dilution of these relationships that can occur at the group level. Specifically, we explored regional *C9orf72* expression across cortical, subcortical, and cerebellar regions using correlation network analysis.

### *C9orf72* co-expression patterns in the brain

**FIGURE 3:**
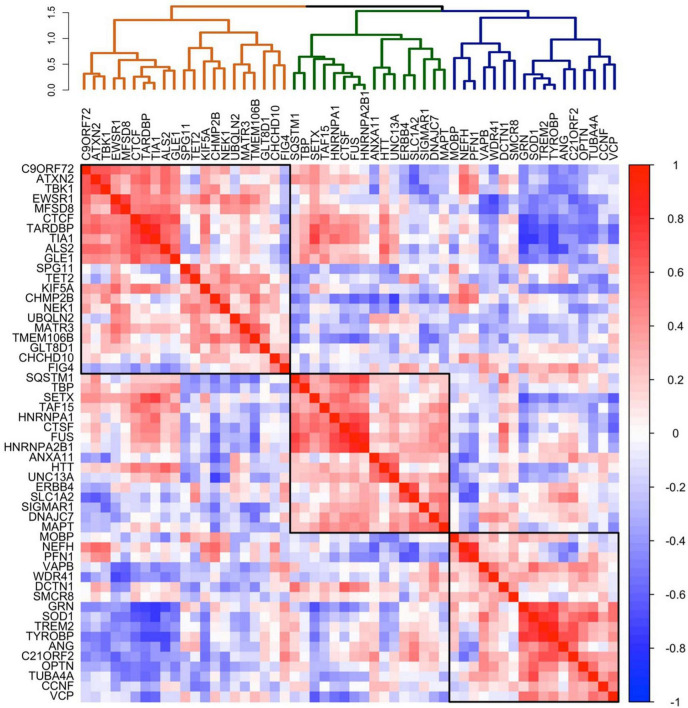
Co-expression of selected 54 ALS-FTD genes in the brain. Co-expression patterning for the expression of selected 54 ALS-FTD genes. The complete linkage method was used to identify 3 clustering groups (black squares).

To explore whether the patterns of *C9orf72* mRNA expression in brain resemble the patterns of mRNA expression of other well-established genes known to cause ALS, FTD, or combined ALS-FTD, we correlated the average *C9orf72* mRNA expression values across the whole brain (51 brain regions) with average mRNA expression values across the whole brain for 54 ALS/FTD associated genes (Table 1). As shown in the dendrogram in Figure 3, we identified three main clusters: an orange cluster with 21 gene members, a green cluster with 16 gene members, and a blue cluster with 17 gene members. *C9orf72* was in the orange cluster and grouped with 9 other gene members: *ATXN2, TBK1, EWSR1, MFSD8, CTCF, TARDBP, TIA, ALS2*, and *GLE1. C9orf72* mRNA expression across the brain most strongly correlated with *ATXN2* (bicor = 0.67) and *TBK1* (bicor = 0.66) mRNA expression. Interestingly, as shown in the 54 × 54 gene co-expression matrix, the orange cluster was anti-correlated with several gene members from the green and blue clusters, including *GRN, TREM2, TYROBP, OPTN, SOD1, ANG, CCNF, C21ORF2*, and *VCP*, among others. Overall, the strongest positive correlations in the 54 × 54 gene correlation matrix were between *TREM2* and *TYROBP* (bicor = 0.87), *HNRNPA2B1* and *FUS* (bicor = 0.86), and *TARDBP* and *TIA* (bicor = 0.83). The strongest negative correlations were between *GRN* and *TIA* (bicor = −0.75), *SOD1* and *TIA* (bicor = −0.74), and *TARDBP*, and *GRN* (bicor = −0.74). The full results can be found in Supplementary Table 2.

### Independent sample validation

To independently validate the correlation patterns of the 54 specified genes associated with ALS/FTD, we compared gene co-expression in the AHBA dataset with the GTEx dataset using a Mantel test (see “Materials and methods”) (Supplementary Figure 1). The results indicated a statistically significant correlation between the gene co-expression patterns in the two datasets (*bicor* = 0.16, *p* = 0.0001) (Supplementary Figure 2). Notably, in the dendrograms generated from both datasets, *C9orf72, TBK1*, and *ATXN2* formed a tight-knit cluster at the lowest of branch heights, suggesting greatest similarities of gene expression between these genes across both datasets. These results add a layer of confidence to the findings, reinforcing the conclusion that the observed similarity in co-expression is likely not a random occurrence.

### Supporting evidence for correlated gene expression and potential pathogenic roles

By testing a large number of genes and correlations it is possible to create a complex dataset that is open to multiple interpretations. Therefore, to strengthen the connection between correlated gene expression and potential pathogenic roles, we explore whether genes associated with specific neurodegenerative diseases, such as *SMCR8* and *WDR41*, known to form a complex with *C9orf72* as part of normal cellular physiology, and *TREM2*, *TYROBP*, and *GRN* associated with leukoencephalopathy and FTD, show correlated gene expression. These analyses revealed intriguing patterns (Supplementary Figure 3): While *C9orf72* negatively correlated with *WDR41* (*bicor* = −0.52), this result did not achieve statistical significance following permutation analysis (*p*-value = 1.00). No significant correlation was found between *C9orf72* and *SMCR8*. However, *SMCR8* and *WDR41* were positively correlated (*bicor* = 0.33, *p*-value = 0.005). Additionally, *GRN, TREM2*, and *TYROBP* displayed strong positive correlations among themselves (bicor ≥ 0.65, *p*-value = 0.0001). These relations were preserved in the larger AHBA 54 × 54 ALS/FTD correlation matrix (Figure 3), where *GRN, TREM2*, and *TYROBP* formed a closely-knit cluster, while *SMCR8* and *WDR41* closely clustered. Further, these relations were generally maintained in GTEx, with the exception of *GRN*, which integrated into a larger cluster alongside *TARDBP, C9orf72*, and other genes (Supplementary Figure 1).

### *C9orf72* co-expression patterns across different anatomical divisions

**FIGURE 4:**
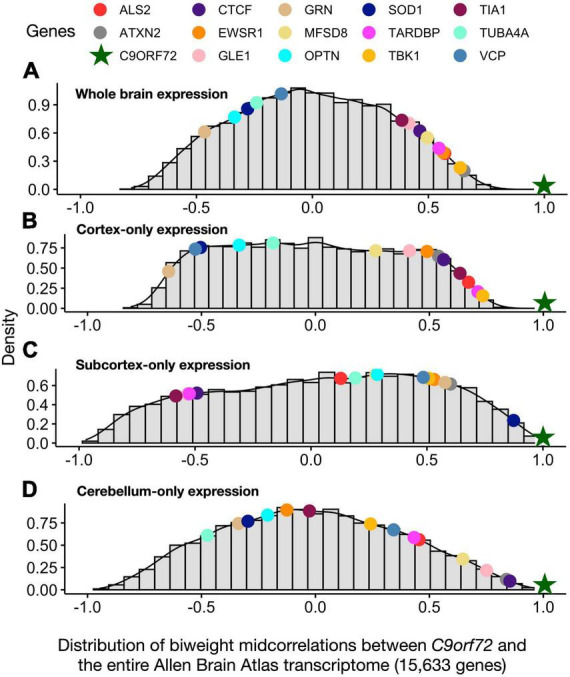
Distribution of biweight midcorrelations between *C9orf72* and all genes in the Allen Human Brain Atlas across different anatomical distributions. **(A)** whole brain **(B)** cortex-only **(C)** subcortex-only, and **(D)** cerebellum-only. Biweight midcorrelations correlations are visualized on a density distribution.

We used the same data driven approach described above to screen for new genes that were co-expressed with *C9orf72*, beyond the 54 known ALS/FTD genes. Thus, we correlated the average *C9orf72* mRNA expression values across the whole brain with average mRNA expression values for each of the 15,633 protein-coding genes. Then, to determine whether the whole brain co-expression patterns were driven by gene expression differences between the anatomical subdivisions, we visualized the similarities in whole brain co-expression and expression patterns among the different anatomical subdivisions (Figures 4A–D). Visualizing the correlation values between *C9orf72* and a few gene members from the orange, green, and blue clusters (Figure 2) revealed that patterns across the whole brain most closely resemble the same relationships within cortical areas. Thus, for the imaging transcriptomic analyses described below, we focus on *C9orf72-*co-expressed genes, defined as genes with a correlation value higher than the absolute value of 0.5 across one or more anatomical subdivisions (*n* = 9,789 genes). The full results can be found in (Supplementary Table 3).

### Imaging transcriptomics analyses: correlation between *C9orf72* co-expressed genes and cortical thickness in symptomatic *C9orf72* HRE carriers

To identify a network of genes that may contribute to the regional vulnerability of the human brain to *C9orf72* HRE*-*mediated pathology, we calculated correlations between average mRNA expression for each of the *C9orf72-*co-expressed genes from AHBA and differences in average cortical thickness from symptomatic *C9orf72* HRE carriers compared to controls (Figures 1E–G). Both brain mRNA expression values from the AHBA dataset and brain imaging measures from the *C9orf72* HRE carriers and controls were mapped to the FreeSurfer average cortical surface, allowing for these imaging transcriptomic correlations.

**FIGURE 5:**
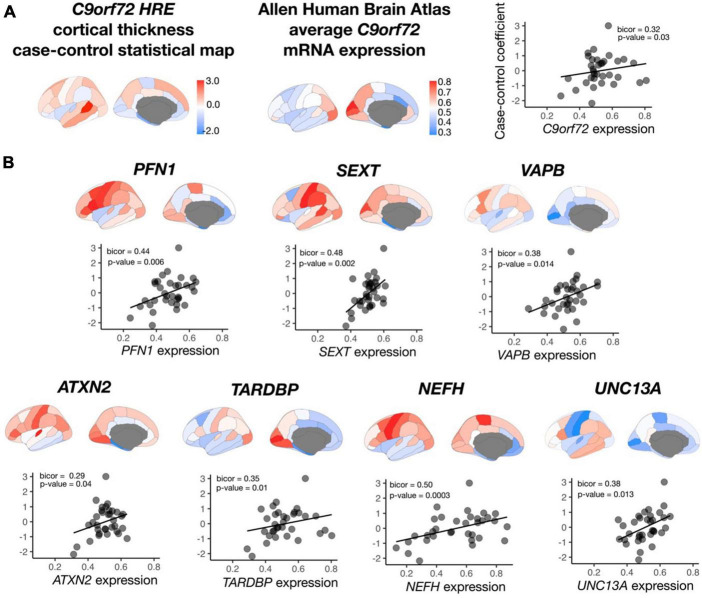
Correlation between *C9orf72* co-expressed genes and cortical thickness coefficients from symptomatic *C9orf72* HRE carriers compared to controls. **(A)** case-control cortical thickness coefficients, average *C9orf72* mRNA expression from Allen Human Brain Atlas in the same regions, and correlation between the two (top panel). **(B)** In addition to *C9orf72*, average mRNA expression in 7 out of 15 ALS/FTD genes significantly correlated with case-control cortical thickness coefficients.

After permutation testing (*p*-value < 0.05), average mRNA expression from roughly 20% of all *C9orf72-*co-expressed genes (*n* = 2,120 genes) significantly correlated with average cortical thickness in symptomatic *C9orf72* HRE carriers, including *C9orf72* and 7 out of the 54 ALS/FTD genes: *TARDBP*, *ATXN2, NEFH*, *SETX*, *PFN1*, *VAPB*, and *UNC13A* (Figure 5). Other correlations within the top 10 most significant include *PLEKHH3, RGS14, LRCH1*, and *PCDHB10*, notable for their prior implication in dementia and aging (Supplementary Table 4).

### Evaluation of cell populations within the brain

**FIGURE 6:**
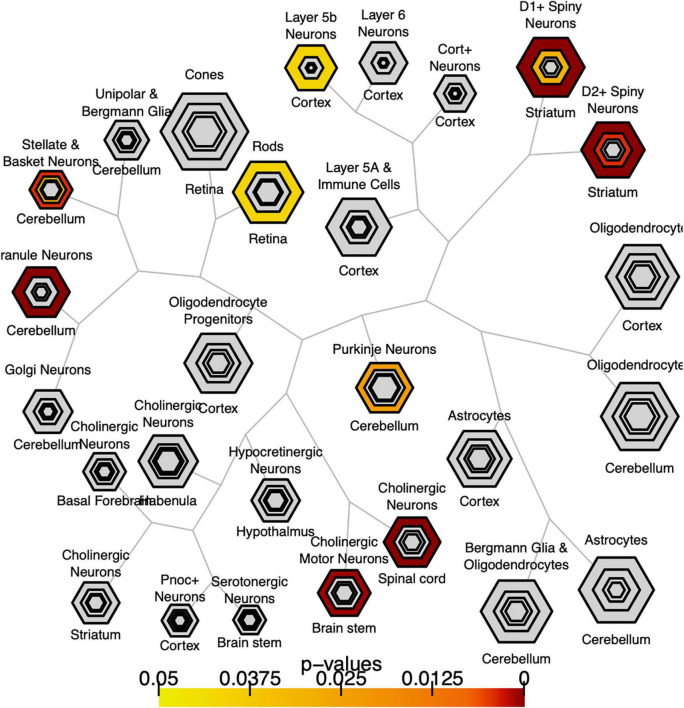
Bulls eye plot showing specific expression analysis across cell types (CSEA) of *C9orf72*-associated genes. Output of CSEA reveals a substantial over-representation at specificity index threshold (pSI) level (*p*-values 0.05) for layer 5b neurons, cholinergic motor neurons, and striatal medium spiny neurons. Bonferroni-Hochberg values are plotted by color.

We explored whether genes with significant *C9orf72* case-control imaging transcriptomic correlations (*n* = 2,120 genes; henceforth, “*C9orf72* imaging transcriptomic genes”) from the previous analyses were enriched for different cell populations in the brain. To do this, we used the publicly available Cell-type Specific Expression Analysis (CSEA) tools, which extracts data from the Brainspan database.^6^ These genes were enriched for several cell types or systems previously implicated in ALS and FTD (Figure 6) (BH adjusted *p*-value < 0.05), including layer 5b cells (Genc et al., 2017; Nana et al., 2019; Vatsavayai et al., 2019), cholinergic motor neurons in the spinal cord and brainstem (Casas et al., 2013), and dopamine type 1 and 2 receptor-positive (Drd1 + , Drd2 +) medium spiny neurons of striatum (Riku et al., 2016; Sobue et al., 2018).

### Identification of *C9orf72*-associated enriched KEGG pathways and GO terms

**FIGURE 7:**
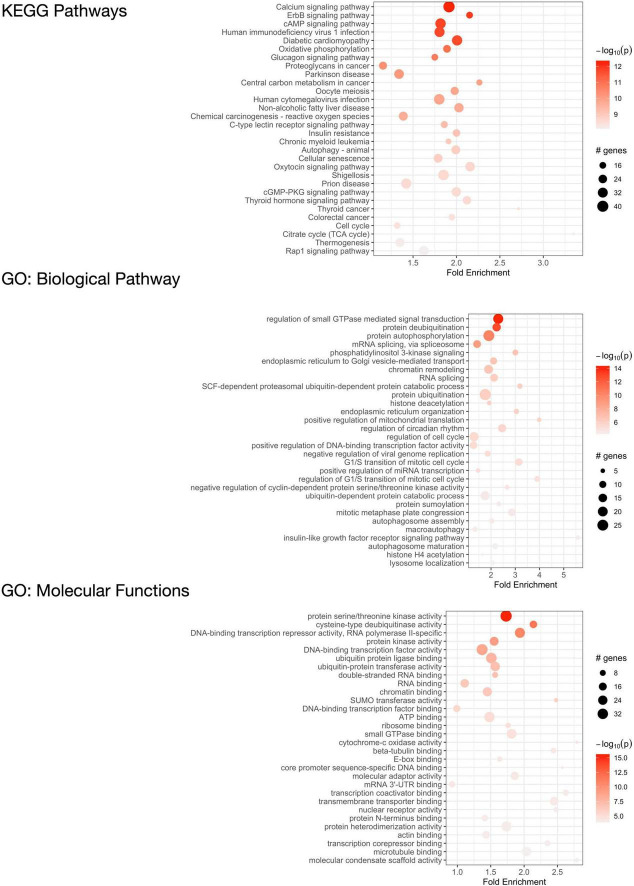
Functional enrichment analysis of *C9orf72*-associated genes. KEGG pathways that are significantly enriched in 2,120 significant *C9orf72* imaging transcriptomic genes (top) and Gene Ontology (GO) analysis of biological processes (middle) and molecular functions (bottom).

We performed pathway enrichment analysis using the R statistical package “pathfindR” (Ulgen et al., 2019) to identify the biological pathways and mechanisms that the 2,120 *C9orf72* imaging transcriptomic genes were enriched for (Figure 7, Supplementary Table 5). The most relevant KEGG biological pathways included autophagy; the Rap1, CAMP, and ErbB signaling pathways; and additional neurogenerative diseases (i.e., Parkinson’s disease and prion disease). Further, the most relevant GO biological pathway terms included mRNA splicing, endoplasmic reticulum to Golgi vesicle-mediated transport, protein ubiquitination, cellular response to DNA damage, regulation of miRNA transcription, and macroautophagy. Between KEGG pathways and GO biological pathway terms, over 25 genes were driving the autophagy enrichment, including *C9orf72.* Lastly, the most relevant GO molecular function terms included DNA binding transcription factor activity, RNA and microtubule binding, and protein ubiquitination. The full list of pathways and corresponding genes can be found in Supplementary Tables 5.

## Discussion

We leveraged mRNA expression data from the AHBA and brain imaging data from 19 *C9orf72* HRE carriers to identify a network of genes and gene products that might contribute to the regional vulnerability of human brain to *C9orf72*-mediated pathology and, more broadly, to ALS and FTD. A total of 2,120 genes showed similar anatomical distribution of gene expression in the brain as *C9orf72* and significantly correlated with patterns of cortical thickness in *C9orf72* HRE carriers compared to controls. These genes were enriched in expression in cell populations previously implicated in ALS and FTD, including layer 5b cells and cholinergic motor neurons in the spinal cord and brainstem, and spiny medium neurons of the striatum. Despite there not being a one-to-one correspondence between gene expression levels and resultant protein levels in patients with different neurodegenerative conditions, we began to probe the potential functional relevance of the *C9orf72* imaging transcriptomics gene network by leveraging known protein-protein interaction networks to identify enriched pathways. The *C9orf72* imaging transcriptomic gene network was enriched for biological and molecular pathways associated with autophagy, mRNA splicing, endoplasmic reticulum to Golgi vesicle-mediated transport, protein ubiquitination, and cellular response to DNA damage, among others. Considered together, we identified a network of *C9orf72* associated genes that may influence selective regional and cell-type-specific vulnerabilities in ALS/FTD.

Different brain regions have distinct distributions of cell types that are segregated into layers and have shared and unique gene expression profiles. Ultimately, these cell types form functional circuits that support motor and cognitive function, language, and social behavior. The identified 2,120 *C9orf72* imaging transcriptomic genes from our analyses showed selective expression in cell types previously implicated in the ALS/FTD-spectrum, including layer 5b cells in the cortex and cholinergic motor neurons in the spinal cord and brainstem (Figure 6). Betz cells, which are upper motor neurons, are found in layer 5b of the motor cortex. They are the largest cells in the neocortex and support long-range cortico-motor neuronal projections by sending their axons down to the spinal cord via the corticospinal tract, where they synapse directly with cholinergic lower motor neurons of the anterior horn of the spinal cord, which in turn synapse directly with their target muscle (Genc et al., 2017; McColgan et al., 2020). Damage to this system and degeneration of Betz cells and cholinergic lower motor neurons, are pathological hallmarks of ALS (Cleveland and Rothstein, 2001; Casas et al., 2013). Also located in layer 5b, but in anterior cingulate and fronto-insular cortices, are von Economo neurons (VENs) and fork cells (Nana et al., 2019; Vatsavayai et al., 2019). VENs and fork cells are selectively vulnerable to bvFTD. The anterior cingulate and fronto-insular cortices are key regions that support social-emotional functions (Seeley et al., 2007; Uddin, 2015; Lin et al., 2019). These regions are the earliest and most consistently affected in patients with sporadic bvFTD (Kril and Halliday, 2004; Seeley et al., 2008). Also, within layer 5b of anterior cingulate and frontoinsular cortices in brain tissue of FTD patients, VEN and fork cells show disproportionate tau aggregation in FTLD-tau (Lin et al., 2019) and TDP-43 aggregation in FTLD-TDP (Nana et al., 2019; Vatsavayai et al., 2019), suggesting that VEN and fork cell biology are key aspects of FTD pathobiology. Furthermore, the *C9orf72* imaging transcriptomic genes were selectively expressed in medium spiny neurons of striatum. Atrophy in striatum, including caudate and nucleus accumbens, is another key feature in the ALS/FTD patients with behavioral and cognitive abnormalities, with and without *C9orf72* mutations (Masuda et al., 2016; Mirzaeva et al., 2016; Sobue et al., 2018). Corroborating these findings, previous studies have found that patients with ALS/FTD show markedly reduced striatal medium spiny neurons, particularly in the caudate head (Riku et al., 2016, 2017). In line with this work, the present study found that the caudate was the most significant region for *C9orf72* expression - *C9orf72* expression was lower here (Figure 2). Taken together, we identified a set of *C9orf72* imaging transcriptomic genes with selective expression in cell types known to be affected in ALS/FTD. Altering the normal gene expression profile of genes comprising the *C9orf72* imaging transcriptomic network may, in turn, alter the corresponding functional circuits they participate in. Thus, disruption to one or more of these functional circuits may lead to converging ALS/FTD phenotypes via different paths. Further, given that the caudate was the most significant region for *C9orf72* expression and *C9orf72* expression was lowest, *C9orf72* expression in this region may impact the intensity of *C9orf72*-specific pathological processes.

The *C9orf72* imaging transcriptomic genes were associated with GO biological pathway and molecular function terms in autophagy, endoplasmic reticulum to Golgi vesicle-mediated transport, cellular response to DNA damage, DNA binding transcription factor activity, and protein ubiquitination. These pathways have been consistently associated with ALS/FTD (Serpente et al., 2015; Granatiero et al., 2021; Kallstig et al., 2021; Chua et al., 2022; Ohshiro et al., 2022), and *C9orf72*-ALS/FTD specifically (Serpente et al., 2015; Shao et al., 2020; Beckers et al., 2021). By leveraging protein-protein interaction information, we found that *C9orf72* was associated with autophagy along with over 25 other genes. These findings have important implications for future research developing molecular-based clinical endpoints for clinical trials. For example, a potential therapeutic strategy that may be applied for the treatment of ALS/FTD is the use of small molecules that can affect autophagy dynamics (Zhang et al., 2021). Since *C9orf72* and these genes are involved in the enriched term, developing a novel protein-based biomarker that incorporates their levels may help capture risk for ALS/FTD. Further, since cortical patterns of mRNA regional expression of the autophagy enriched genes were strongly correlated with cortical thickness patterns in symptomatic *C9orf72* HRE carriers, it may be worthwhile to develop multimodal biomarkers based on cortical thickness measures and gene or protein expression data.

Brain imaging allows for non-invasive visualization and monitoring of structural and functional changes, aiding in early diagnosis, understanding disease progression, and facilitating the development and assessment of treatment interventions in neurodegenerative diseases. Advancements in high-throughput tissue processing enabled the creation of comprehensive transcriptomic atlases for the brain. By combining imaging and transcriptomic information, imaging transcriptomics aims to provide a comprehensive understanding of how genetic factors contribute to brain structure and function and offering insights into the molecular underpinnings of neurodegeneration. To date, the Allen Human Brain Atlas (AHBA) is the most anatomically comprehensive gene expression atlas. In the past decade, several studies have leveraged AHBA database to illuminate potential pathophysiological mechanisms in neurodegeneration [for a recent review see (Arnatkeviciute et al., 2022)]. A recent study explored co-expression patterns associated with brain atrophy patterns in symptomatic FTD mutation carriers in *C9orf72*, *GRN*, and *MAPT* and report top 20 genes and relevant biological pathways (Altmann et al., 2020). Of these, we identified four genes, *KATNAL2, ABRACL, FXR1*, and *HIST1H4A*, that were reported as most significant in *C9orf72* HRE carriers. Our study differs from this one in that we investigated co-expression patterns specifically related to cortical thickness, as opposed to focusing on brain atrophy, and our brain maps control for age and sex. Another study set out to determine the correlation between regional brain metabolism using fluorodeoxyglucose PET and regional expression of Alzheimer-risk genes in the AHBA. Regional brain expression of several Alzheimer-risk genes, including *APOE*, *CD33*, and *SORL1*, showed a strong correlation with brain metabolism, particularly in regions of the brain that are affected earliest and most severely in Alzheimer’s disease (Ye et al., 2022). Taken together, better understanding the relationship between gene expression and imaging phenotypes in neurodegenerative conditions may illuminate potential pathophysiological mechanisms that contribute to disease susceptibility.

There are limitations to this study. Gene expression data was available using brain tissue data from healthy individuals. Therefore, the regional gene expression and imaging correlations described herein represent the relationship between regional gene expression levels in the healthy human brain and regional cortical thickness in symptomatic *C9orf72* HRE carriers compared to controls. Future studies will be needed to assess regional gene expression and imaging data from the same participants. Also, research has shown that in the frontal cortex alone, thousands of genes are differentially expressed in *C9orf72* HRE carrier ALS-FTD patients versus ALS-FTD patients without *C9orf72* HRE (Dickson et al., 2019), and distinct expression patterns were evident in *C9orf72* HRE carriers compared to healthy controls. In our imaging transcriptomics analyses, all imaging data were sourced exclusively from individuals diagnosed with ALS, FTD, or ALS-FTD who are *C9orf72* HRE carriers, as well as from healthy controls. Therefore, an important limitation is that our results may not generalize to individuals with ALS/FTD without *C9orf72* HRE. Interpretation of our imaging transcriptomics study is, therefore, constrained to regional patterns of gene expression in the healthy brain mirroring regional patterns of cortical thickness in *C9orf72* HRE carriers compared to controls. An additional limitation to transcriptomic studies more broadly is that we cannot assume a one-to-one correspondence between gene expression levels and resultant protein levels. In our analyses, *TREM2, TYROBP*, and *GRN* associated with leukoencephalopathy and FTD, show correlated gene expression, however, *SMCR8* and *WDR41*, known to form a complex with *C9orf72*, did not. Therefore, gene expression changes in *C9orf72* due to the HRE represents only a partial understanding of the underlying pathogenic processes. Lastly, we acknowledge that additional insights could be gained by knowing if any of the six brain donors were carriers of the *C9orf72* intermediate allele/expansion-associated haplotype (e.g., rs2814707 T allele) since it is common (MAF ∼20% in EUR) and has been shown to significantly affect the expression levels of *C9orf72*. However, genotype information for the donors is not available.

## Conclusion

We explored the neuroanatomical basis of shared genetic risk in the ALS/FTD spectrum by identifying genes that show regional co-expression patterns similar to *C9orf72*, the most common genetic contributor to ALS and FTD. In doing so, we identified a *C9orf72* imaging transcriptomic gene network that was enriched in cell populations and regions within the brain known to be selectively vulnerable to ALS/FTD. Altering the normal gene expression patterns of these *C9orf72* imaging transcriptomic genes may disrupt cell-to-cell communication and protein-protein interactions between the specific cell-types/brain regions and, in turn, render these cell-types/brain regions vulnerable to ALS/FTD pathobiology. Future work will be required to clarify the impact of disease neuropathology on these neuroanatomical gene expression patterns.

## Data availability statement

Publicly available datasets were analyzed in this study. This data can be found here: https://portal.brain-map.org.

## Ethics statement

The studies involving humans were approved by the UCSF Committee on Human Research. The studies were conducted in accordance with the local legislation and institutional requirements. Written informed consent for participation in this study was provided by the participants’ legal guardians/next of kin.

## Author contributions

IB: Conceptualization, Formal analysis, Funding acquisition, Investigation, Methodology, Resources, Visualization, Writing – original draft, Writing – review & editing. DS: Conceptualization, Formal analysis, Writing – original draft. RN: Visualization, Methodology, Writing – review & editing. LB: Data curation, Formal analysis, Writing – review & editing. SL: Data curation, Funding acquisition, Resources, Writing – review & editing. BM: Data curation, Funding acquisition, Resources, Conceptualization, Writing – review & editing. PC: Conceptualization, Methodology, Writing – review & editing. VS: Conceptualization, Writing – review & editing. LS: Conceptualization, Methodology, Writing – review & editing. RD: Conceptualization, Funding acquisition, Resources, Writing – original draft. JY: Conceptualization, Methodology, Resources, Supervision, Writing – review & editing.

## Footnotes

1
http://human.brain-map.org

2
https://community.brain-map.org/c/how-to/15

3
https://abagen.readthedocs.io/en/stable/

4
https://surfer.nmr.mgh.harvard.edu

5
https://www.diedrichsenlab.org

6
http://genetics.wustl.edu/
