# Supplementary figures and images for: C9orf72 gene networks in the human brain correlate with cortical thickness in C9-FTD and implicate vulnerable cell types
Source: Front Neurosci. 2024 Feb 26;18:1258996. doi: 10.3389/fnins.2024.1258996 (PMC10925697; doi:10.3389/fnins.2024.1258996)

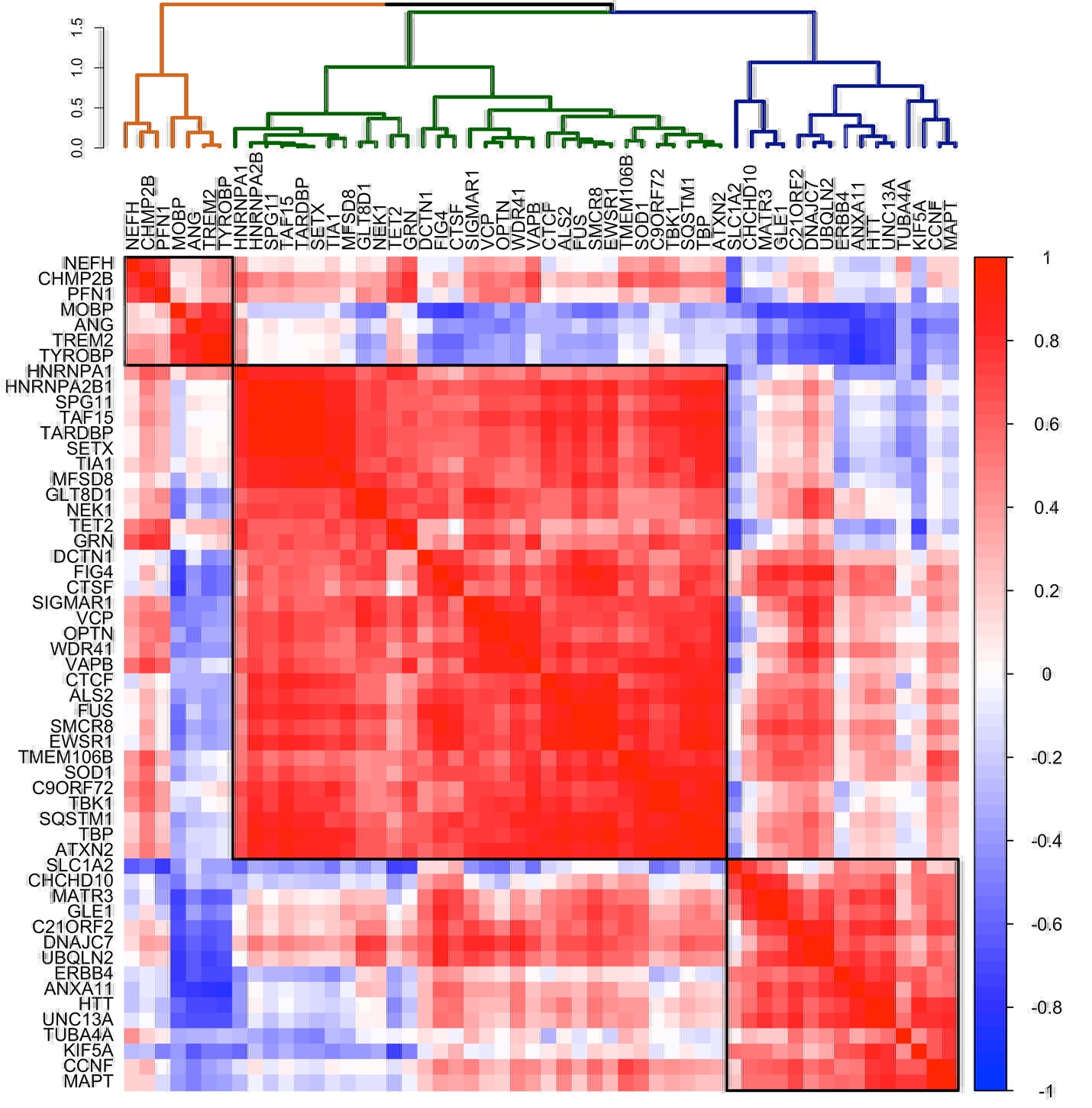

Supplement: Supplementary file 2 [file Image_1.JPEG]

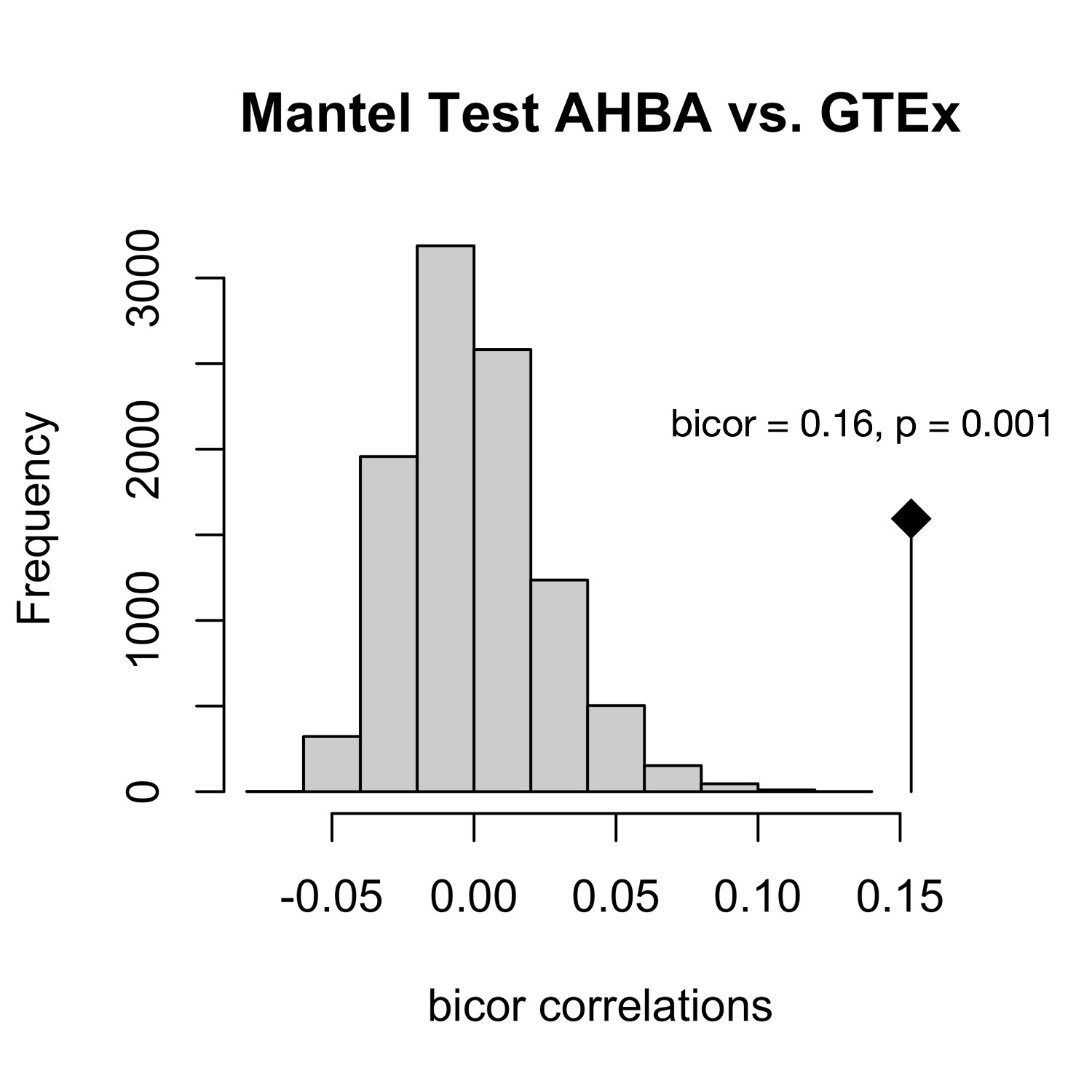

Supplement: Supplementary file 3 [file Image_2.JPEG]

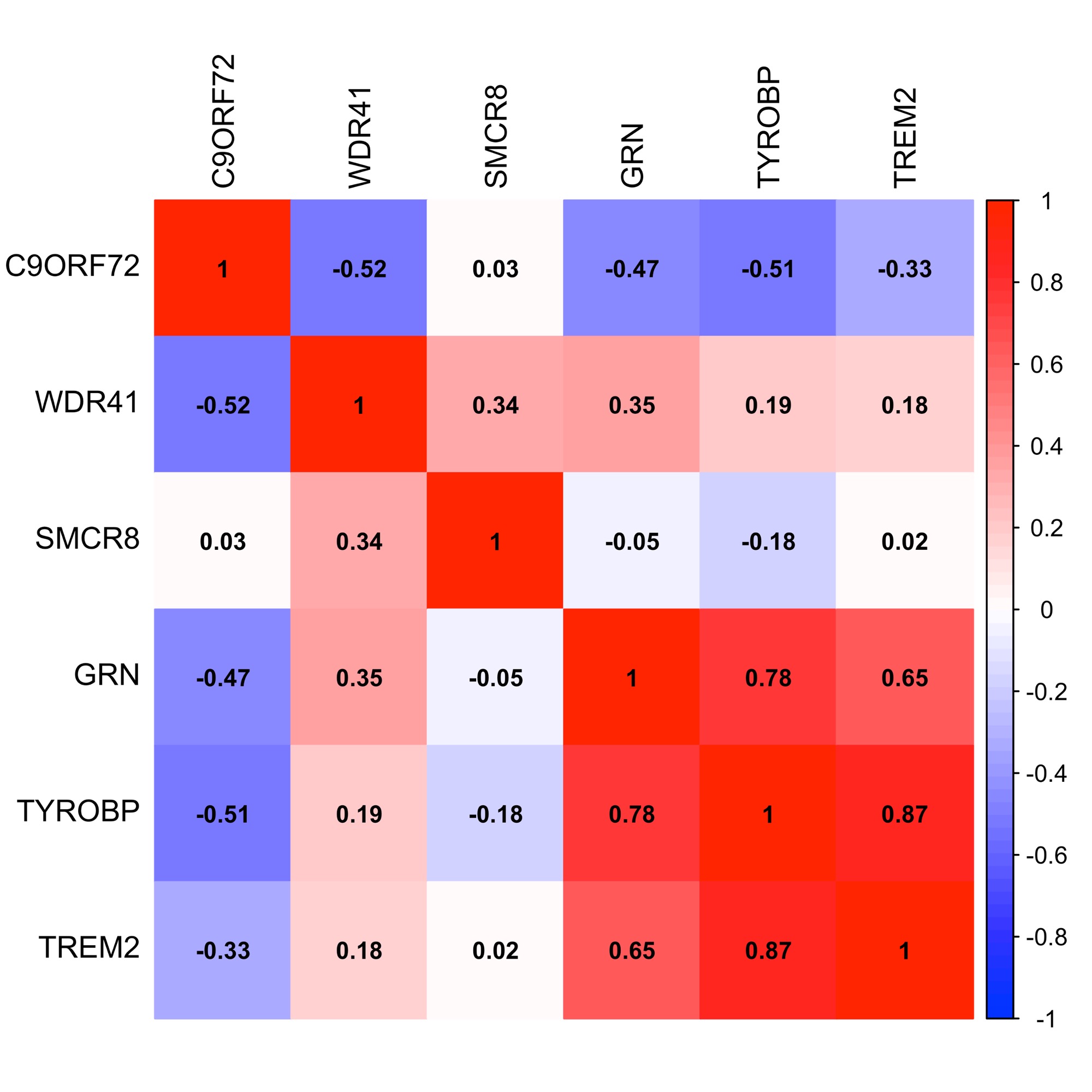

Supplement: Supplementary file 4 [file Image_3.JPEG]
